# What people really change after genetic testing (GT) performed in private labs: results from an Italian study

**DOI:** 10.1038/s41431-021-00879-w

**Published:** 2021-04-12

**Authors:** Serena Oliveri, Clizia Cincidda, Giulia Ongaro, Ilaria Cutica, Alessandra Gorini, Francesca Spinella, Francesco Fiorentino, Marina Baldi, Gabriella Pravettoni

**Affiliations:** 1 Applied Research Division for Cognitive and Psychological Science, European Institute of Oncology (IEO), IRCCS, Milan, Italy; 2 Department of Oncology and Hemato-Oncology (DIPO), University of Milan, Milan, Italy; 3 GENOMA Group, Molecular Genetics Laboratory, Rome, Italy

**Keywords:** Genetic testing, Human behaviour

## Abstract

Despite the widespread diffusion of direct-to-consumer genetic testing (GT), it is still unclear whether people who learn about their genetic susceptibility to a clinical condition change their behaviors, and the psychological factors involved. The aim of the present study is to investigate long-term changes in health-related choices, individual tendencies and risk attitudes in an Italian sample of GT users. In the context of the Mind the Risk study, which investigated a sample of Italian adults who underwent GT in a private laboratory, 99 clients participated in the follow up assessment. They completed a self-administered questionnaire investigating: (a) clinical history and motivation for testing, (b) lifestyle and risk behaviors, (c) individual tendencies toward health, and (d) risk-taking attitude and risk tolerance. Such variables were measured at three different time-points: T0—before GT, T1—at 6 months after genetic results, and T2—at 1 year from results. Results showed that, at baseline, participants who stated they intended to modify their behavior after GT results, effectively did so over time. This result held both for participants who received a positive or negative test result. In general, a healthier diet was the most frequently observed long-term behavioral change. As regards psychological variables, a risk-taking attitude and risk tolerance did not seem to affect the decision to change the lifestyle. Finally, we found an overall reduction in anxiety and worry over health over time, but also a reduction in the motivation for health promotion and prevention, health esteem, and positive expectations for their health in the future.

## Introduction

Direct-to-consumer genetic testing (DTC-GT), involving the use of over-the-counter tests, which are analyzed in private laboratories and results are returned to users directly without the mediation of a healthcare provider, was initially conceived and practiced in the US. Following the release of the first genetic-testing kit by the genomic and biotechnology company 23andMe in 2007, direct-to-consumer DNA testing became object of discussion. The public opinion was soon split between those in favor, exalting its usefulness in terms of preventive decisions, autonomy and empowerment for citizens in managing their health, and those against, emphasizing the risks of such approach, including the potential to cause harm, anxiety, and increased use of unnecessary and expensive screening and medical procedures [1–3]. Due to these concerns, the US Food and Drug Administration (FDA) called upon the 23andMe company in December 2013 to stop marketing its genome-wide SNP testing “Personal Genome Service,” which returned information for a multitude of disease-related SNPs. However, over recent years, the FDA has allowed the marketing of tests that provide genetic risk information for certain conditions (ten different kinds of diseases) [2]. At present, the vast majority of the available literature concerning DTC-GT refers to the US context and largely reflects the perspective of people who have never purchased a DTC-GT [4, 5], or early adopters comprising well-educated people within the scientific community (engineers, biologists, health, and technology experts) who are knowledgeable about techno-scientific innovations [6–8]. In the last 10 years, genetic tests, which are marketed directly to the community, have made their appearance worldwide, spreading to several countries and cultures and also establishing themselves among European users—who are largely different from the US “early adopters” in terms of scientific literacy and background [9]. Prior to this, DTC-GT had been rather unknown in Europe due to several reasons, including the lack of EU or national legislative regulations specifically related to DTC-GT [10], and the custom to restrict access to genetic tests to expert physicians/professionals.

As genetic testing (GT) became increasingly available to the public, one of the questions that arose within the scientific community was: why should people be interested in undergoing GT [11–13]. The hypothesis and sometimes evidence, was that a negative test result could eliminate the need for unnecessary checkups and screening tests, and that a positive test result could help people make better informed decisions on how to manage their health. Testing also provides individuals with a reason to avoid or cope with manageable factors in order to counterbalance their risk through screening and treatment options [14]. On the other hand, carrier disease screenings can help people make decisions about having children [15, 16].

Nevertheless, awareness of health management possibilities does not automatically translate into awareness of health responsibility, important life decisions, or lifestyle changes. A recent meta-analysis [17] showed that, when GT is offered directly to consumers without additional lifestyle counseling, the effects on behavioral changes are modest, even if one quarter of the overall DTC-GT users reported that they made at least one “positive” change in their health behavior (diet, exercise, and sharing results or checkups with clinicians). However, the studies considered in this meta-analysis did not sketch a clear picture of long-term behavioral changes: only a few studies considered the psychological variables that might influence the way people react, advanced from genetic results, or followed-up on consumers to monitor their decisions.

As regards factors influencing short-term reactions and changes, several studies have revealed that socio-demographic aspects, such as parenthood, the family or personal history of disease, life experiences, beliefs and individual characteristics (such as internal vs. external attribution of risk) affect the way people understand results and formulate decisions related to their health and risk [13, 18–23]. Individual differences in disease perceptions (seriousness and controllability) influence psychological outcomes following DTC-GT [24], even though, generally speaking, the psychological impact of the genetic test has been widely demonstrated not to be harmful [2, 13, 14, 17].

With regard to the European context and particularly the Italian population, in a preliminary study conducted by Oliveri et al. [19] the general public expressed a wide interest and evaluated DTC-GT as a useful tool for disease prevention. This, despite the worry that GT results could affect their life planning without having relevant clinical utility in some cases [19]. However, Italian citizens demonstrated enormous trust in the paternalistic figure of the doctor who makes all the main decisions concerning patients’ health and the management of data in genetics [19]. Wöhlke et al. compared Italian and German samples of GT users [25], showing that Italians are higher value to genetic risk information in terms of prevention for themselves and their family. Italian GT users, more than German users, believed that they could counterbalance a genetic predisposition with preventive measures. Another interesting cultural difference concerned the perception of genetic information as providing certainty, which was supported by about three-quarters of German participants, and to a much lesser degree, by Italian respondents. Thus, Italians seem to not expect certain information from GT results regarding future health conditions, but consider these results as a useful indication to actively face the risk.

The present research is the continuation of a study that began in February 2016 (Mind the Risk Project, see “Funding” and “Materials and methods”) and ended in 2019. In this investigation, a sample of Italian adults underwent GT through a private laboratory. They consented to answering a survey related to their socio-demographic profile, their motivations, health-related habits, health orientation, and psychological tendencies. Extensive literature, together with the specific purposes of this investigation and the main results at baseline (the time before undergoing GT), were already described in two recently published contributions [26, 27]. Results described in Oliveri et al. [26] showed that Italian GT users were overall well-educated and predominantly female (82.2%—reflecting the fact that (i) most participants were requesting analyses for BRCA1/2 or fertility problems or multiple miscarriages, and (ii) males usually have higher privacy concerns hindering their availability to participate in this kind of survey). In addition, physicians were their main source of information and managed their genetic results. Taken together, these results stress the importance of the relationship between doctor and patient in the Italian culture, and suggest that in Italy the DTC-GT pure phenomenon is less widespread compared to the US context. We should consider that, over time, the pure model of DTC-GT evolved in different formats, with companies/labs offering services, which span from optional genetic counseling to tests solely delivered through licensed (i.e., physicians order the kits from the company and distribute them to their patients). For this reason, Prainsack and Vayena [28] suggest that, rather than referring to a single “DTC model” it would be more appropriate to talk about a cluster of practices under the label of “beyond-the-clinic” genomics.

Finally, results from Oliveri et al. [26] reported that the majority of participants had a family history or personal experience with a disease and consequently were willing to look for a clinical answer in their genetic makeup. Genetic screening was considered useful for adopting behaviors that may prevent disease onset, for knowing the “real health status,” for adopting health-related behaviors and for motivating a change in behavior after results (healthier diet, exercise and medical checks).

In Oliveri et al. [27], further analysis related to the psycho-decisional profile of the Italian sample of GT users was described, as well as the interactions on the self-perception of poor health and screening habits. A family history of disease resulted to be more likely to determine the intention for lifestyle changes after receiving genetic risk information [27]. From a psychological perspective, our participants were motivated to preserve their well-being; they felt responsible for their health, they were neither pessimistic nor optimistic toward negative occurrences, and they were scarcely inclined to take high risks in their lives. Participants who had previously suffered from a disease tended to be less tolerant of the uncertainty of future negative events, and consequently were more likely to seek genetic risk information to reduce this uncertainty and counterbalance their risk.

This research aims to investigate experiences and decisions over time (after GT results) in this sample of Italian GT users. This article also describes long-term changes in health-related choices, individual tendencies, risk attitudes, and preferences.

Results from this investigation may provide relevant information to stakeholders, in order to truly enhance individual empowerment toward genetic risk information management.

## Materials and methods

### Participants

One hundred and fifty-two clients of GenomaLab, a private laboratory located in Rome and Milan, who underwent a genetic test or panel, were invited and agreed to participate in the Mind the Risk International Study. Mind the Risk is a joint European research program dedicated to the investigation of the psychological and social implications of providing genetic risk information (Mind the risk—ethical, psychological, and social implications of provision of risk information from genetic and related technologies. A joint European research program, funded by The Swedish Foundation for Humanities and Social Sciences (Grant No. M13-0260:1)). As stated in the introduction, the socio-demographic results at baseline of this sample of Italian GT users and their psychological profile, motivations and health orientation, were already discussed in previous published contributions [26, 27].

Among the 152 clients enrolled at baseline, ninety-nine participants completed the follow-up evaluations (response rate 65.1%) investigated in this contribution. The final cohort of 99 participants was mainly composed of females (87.9 %, *f* = 87; *m* = 12), with an age ranging from 18 to 68 years (mean = 42.05; SD = 11.31). Overall recruitment lasted from February 2016 to September 2017 and the follow up from August 2016 to September 2018. Each participant’s data were anonymized and associated with an ID code. The same code was associated with the genetic analysis performed in order to enable pairing with the results received by participants. For analysis purposes, we defined genetic results as “positive” when a variant for a particular clinical condition was detected and “negative” when the investigated variants were not present.

Description of the lab, procedure, measures, and data analysis was moved to the Supplementary Material.

## Results

### Socio-demographic aspects and clinical aspects at baseline

Table 1 provides a detailed description of the socio-demographic characteristics of the 99 Italian GT users who underwent GT via GenomaLab and completed follow up evaluations.

**Table 1:** Socio-demographic variables.

Socio-demographic variables	*N* (%)
Sex	
Male	12 (12.1%)
Female	87 (87.9%)
Marital Status	
Single	13 (13.3%)
Engaged in a relationship or live-in-partner	26 (26.5%)
Married	57 (58.2%)
Separated/divorced	1 (1%)
Widowed	1 (1%)
Educational level	
No education	1 (1%)
Primary school	2 (2%)
High school	44 (44.5%)
Master degree	41 (41.4%)
Postgraduate	11 (11.1%)
Current employment	
School student	1 (1%)
University student	5 (5.1%)
Not working but looking for a job	4 (4%)
Not working and not looking for a job	3 (3%)
Housewife	6 (6.1%)
Laborer	1 (1%)
Fixed-term work	5 (5.1%)
Office work	40 (40.4%)
Freelance professional	25 (25.3%)
Retired	5 (5.1%)
Manager	2 (2%)
Entrepreneur	2 (2%)
Parenthood	
Yes	47 (47.5%)
No	50 (50.5%)
	Mean (SD)
Age	42.05 (11.31)

At the time of testing, 37.4% were suffering from physical disease and 18.2% stated that they had psychological health issues (anxiety, sleep disorders, or depression). 30.3% had suffered from a disease in the past and more than half of the samples stated that they had experienced an important disease of a significant relative (60.6%). 27.3% acknowledged that they had a hereditary/genetic disease history whereas 36.4% did not have enough information to report the presence of hereditary disease in the family. The majority of participants underwent GT for food intolerance (35.4%), cancer susceptibility (23.2%), and reproductive problems (34.3%) (e.g., infertility or multiple miscarriage); other reasons are shown in Table 2. In particular, the main GT category was related to nutrigenomics, celiac disease, thrombophilia, and BRCA1/2. A total of 61 participants received a positive result (variant detected) whereas 38 received a negative result. Clients were in contact with GenomaLab through their physician (38.4% with the doctor as mediator and 20.2% in direct contact with the lab but with the help of their physicians). 35.4% of participants were in direct contact with the lab, which handled their results and provided its counseling services.

**Table 2:** Reasons for undergoing GT reported by clients.

	*N* (%)
Type of genetic testing	
Food intolerance	35 (35.4%)
Reproductive choice/infertility	34 (34.3%)
Cancer susceptibility	23 (23.2%)
Huntington disease	1 (1%)
Hemocromatosis	1 (1%)
Lipid metabolism	3 (3%)
Macular degeneration (AMD)	1 (1%)
Bone metabolism	1 (1%)
Genetic-testing result	
Positive	61 (61.62%)
Negative	38 (38.38%)

We asked about their main dietary habits and physical activity: the majority of clients (58.2%) reported that they followed a Mediterranean diet, whereas 23.5% followed a vegetarian diet; 21.1% reported that they practised vigorous physical activity, 53.3% moderate physical activity and only 21.1% low physical activity (4.4% did not practise any physical activity). 56.7% of participants had never smoked while 23.2% were former smokers; only 19.6% were currently smokers. This data depict a sample that is really mindful toward an overall healthy lifestyle.

Furthermore, results showed that Italian clients had a low propensity toward very high risks (*M* = 2.58; SD = 1.75) and around half of participants showed an overall low risk tolerance (38.5% very low risk tolerance and 14.3% low risk tolerance). These aspects are also attributable to their tendencies toward health.

### Follow-up comparison of behaviors and GT results, risk tolerance, or overconfidence

At baseline, 71.4% of clients declared their intention to change their lifestyle following GT results, whereas 29.6% stated they had no intention of making changes. At the 6-month follow-up (T1) from results, 50.8% of people who received a positive result reported that they had modified their lifestyle, while the percentage at 1 year was 62.3% (N38) (all statistical values regardless of significance are provided in Table S1). Among people who received negative results, 21% changed their lifestyle after 6 months from the disclosure of results, and the percentage at 1 year was 28.9% (*N* = 11) (Table S1).

A more detailed analysis of the characteristics of subjects who effectively changed something in their lifestyle at 1 year, controlling for the positive/negative result, demonstrated that these were already motivated to change, even before undergoing the blood sample for the genetic analysis (baseline). In fact, they changed their lifestyle independently of whether they received a positive or negative result. Among the 38 subjects who received a positive result and effectively modified their lifestyle at 1 year, 33 subjects also answered “yes” when asked at baseline “Do you think you will change your lifestyle after receiving your genetic test results?”

We considered the interaction between test-type*test-result in order to verify if positive or negative result in a specific type of GT had a higher impact on lifestyle changing after 6 months and 1 year. Contingency tables and Chi-square tests showed that results in food intolerance testing had a significant influence in lifestyle changes at 6 months [*X*^2^ (5) = 17.159; *p* = 0.004)] and after 1 year [*X*^2^ (5) = 21.910; *p* = 0.001)]. As showed in Table 3, at 6 months about 75% of participants who received a positive result for food intolerance changed their lifestyle (adjusted residuals = −3.3), at 1 year they were 87.5% (adjusted residuals = 3.6). Whereas, seven participants over eight who received a negative result did not change anything at 6 month and 1 year (adjusted residuals = 2.6). Contingency table also showed that clients who received negative results at cancer susceptibility testing and decided not to change their lifestyle after 1 year were significantly more than expected (68.8% adjusted residuals = 2.3).

**Table 3:** Contingency table test-type*test-result influencing lifestyle change.

			Change lifestyle post result_T1		Change lifestyle post result_T2		Total
			No	Yes	No	Yes	
Test-type*test-result	Reproductive choice/infertility*negative result	*N* (%)	8 (80)	2 (20)	6 (60)	4 (40)	10 (100%)
		Adjusted residuals	1.8	−1.8	1.1	−1.1	
	Reproductive choice/infertility*positive result	*N* (%)	11 (52.4)	10 (47.6)	7 (33.3)	14 (66.7)	21 (100%)
		Adjusted residuals	−0.1	0.1	−1.1	1.1	
	Cancer susceptibility*negative result	*N* (%)	11 (68.8)	5 (31.3)	11 (68.8)	5 (31.3)	16 (100%)
		Adjusted residuals	1.4	−1.4	2.3	−2.3	
	Cancer susceptibility*positive result	*N* (%)	1 (25)	3 (75)	2 (50)	2 (50)	4 (100%)
		Adjusted residuals	−1.2	1.2	0.3	−0.3	
	Food intolerance*negative result	*N* (%)	7 (87.5)	1 (12.5)	7 (87.5)	1 (12.5)	8 (100%)
		Adjusted residuals	2.1	−2.1	2.6	−2.6	
	Food intolerance*positive result	*N* (%)	6 (25)	18 (75)	3 (12.5)	21 (87.5)	24 (100%)
		Adjusted residuals	−3.3	3.3	−3.6	3.6	
Total		*N* (%)	44 (53)	39 (47)	36 (43.4)	47 (56.6)	83 (100%)

**Fig. 1:**
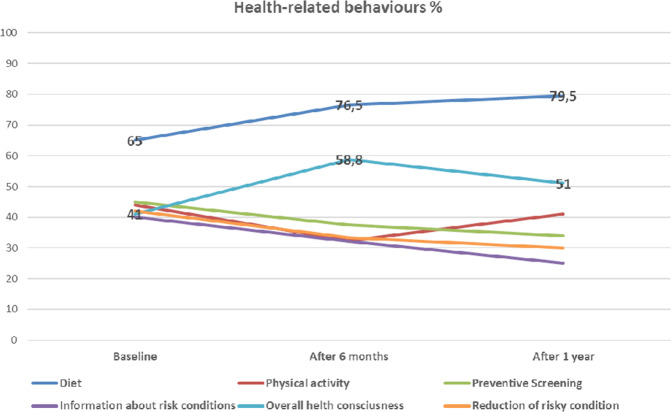
Changes in health-related behaviours over time. Shown in the figure are the percentages of subjects who decided to change specific aspects of their lifestyle at T1 (6 months) and at T2 (1 year).

We asked clients which health-related improvements they wanted to adopt and which they effectively adopted after results. At baseline, the most mentioned changes were a “healthier diet” (65.2%), an increase in “preventive screening” (46.4%) and more “physical activity” (43.5%). The “healthier diet” was the most effectively adopted behavior both at 6 months (76.5%) and at the 1-year follow up (79.5%), followed by an overall higher consciousness of the importance of monitoring their health (see Fig. 1 and Table S1). “Preventive screening” and “physical activity” were not confirmed among the genuine adopted changes.

The result concerning changes in dietary aspects was confirmed by the contemporary overall reduction of dietary risk behaviors over time (such as alcohol or junk food intake) (*F*_(1.530, 148.367)_ = 13.172, *p* < 0.01). In particular, a positive GT result had a significant impact on the reduction of dietary risk behaviors (*F*_(1.561, 149.817)_ = 5.584, *p* = 0.009). Instead, physical activity and preventive screenings were not confirmed as a parameter that GT users actually changed at 6 months (physical activity = 32.4%; medical preventive checkups = 37.5%) and the 1-year follow-up (physical activity = 41%; medical preventive screenings = 34%).

Nevertheless, the majority of our GT users stated that they had regular medical checkups ever since the beginning and this trend did not change over time (baseline = 62.6 %; at 6 months = 62.1%; at 1 year = 65%). We evaluated whether there was a difference in having medical checkups based on the GT result. It interestingly emerged that at T1 (6 months), the group of clients who received a negative result started to have regular medical checkups more frequently than those who received a positive result (*X*^2^ (4) = 11.769; *p* = 0.019). This trend was not confirmed at T2. Furthermore, we evaluated whether there was a difference in medical checkups based on the type of GT performed and no significant changes were found (Table S1).

The contingency tables showed that the groups of clients with different levels of risk tolerance (very low, low, moderate and high) did not significantly differ in their intention to change their lifestyle post results (*X*^2^ (3) = 7.805; *p* = 0.05). However, at 1-year follow-up, the group of participants with very low risk tolerance changed their habits significantly more frequently when compared to all other groups (57,8%) (*X*^2^ (3) = 8.877; *p* = 0.03). In addition, the analysis of standardized residual indicated that the “medium risk tolerance” group opted to change their lifestyle less frequently than expected at T2 (15.6% [*n* = 7] vs. 41.7% [*n* = 15]; standardized residual = 2.6).

Finally, independent sample *t*-tests revealed that overconfidence only influenced participants’ intention to change their lifestyle at baseline (*t* (96) = 2.228, *p* = 0.028), but no effective changes at 6 months: [*t* (85) = 1.297, *p* = 1.98] and at 1 year [*t* (86) = 0.180, *p* = 0.858)].

### Health Orientation Scale (HOS) Italian adaptation [29]

Before testing (baseline), the GT users included in this study showed a high motivation for health promotion and prevention (*M* = 17.40; SD = 3.75), medium levels of anxiety (*M* = 12.73; SD = 3.17), health expectation (*M* = 5.01; SD = 2.67), and health esteem (*M* = 15.58; SD: 2.41).

The repeated measure analysis of variance (ANOVA) showed that all psychological aspects significantly changed over time (*MHPP*: *F*_(1.546, 129.852)_ = 22.441, *p* = 0.000; *HES*: *F*_(1.442,121.165)_ = 30.359, *p* = 0.000; *HA*: *F*_(1.463,122.930)_ = 33.142, *p* = 0.000; *HE:* *F*_(1.301, 109.268)_ = 66.854, *p* = 0.000). In particular, health anxiety, health expectation, health esteem, and motivation for health promotion and prevention had already decreased significantly 6 months after the receipt of GT results (T1) (see Table 4 for mean scores and standard deviations).

**Table 4:** HOS subscales mean scores at T0, T1 e T2.

Subscale	T0 baseline mean (SD)	T1 6-month mean (SD)	T2 1-year mean (SD)	*F*
Health anxiety	12.73 (3.17)	9.95 (3.95)	10.51 (2.68)	22.441**
Motivation to avoid unhealthiness	17.40 (3.75)	15.20 (4.37)	15.42 (4.43)	33.142**
Health esteem and confidence	15.58 (2.41)	12.86 (4.21)	13.03 (4.29)	30.359**
Health expectation	5.01 (2.67)	3.69 (3.64)	3.76 (3.77)	66.854**

**p < 0.01.

The mixed factorial ANOVA confirmed the effect of time in the HOS subscale decrease, but showed that there was no effect from the type of GT performed (GT for intolerance, infertility problems, or cancer susceptibility) or GT results (positive or negative) on such psychological variables over time. The analysis also revealed that effective behavioral changes at 6 months were related to moderate anxiety levels (*t* (85) = −2.886, *p* = 0.012). This means that clients who claimed to have actually changed their lifestyle at T1 had significant higher levels of anxiety (*M* = 11.26; SD = 4.64) than clients who did not make any changes (*M* = 8.90; SD = 2.94).

### Lab contact and decision on sharing results

**Fig. 2:**
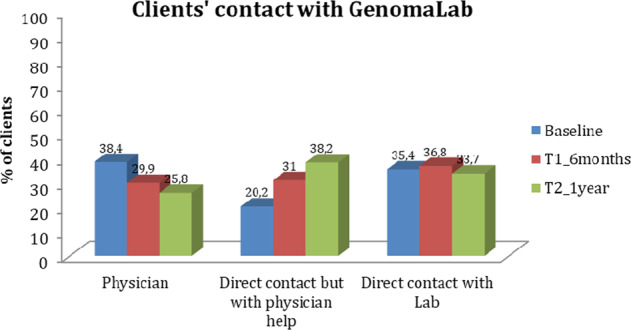
Clients’ contact with GenomaLab. In the figure are reported the modes of contact that clients preferred to keep with the Lab and to maintain for managing their results, and the relative percentage of subjects at baseline, at T1 (6 months) and at T2 (1 year).

Figure 2 reports the percentages of clients who maintained direct contact with the genetic lab, maintained contact through their physicians or left the responsibility for the management of results and contact solely in the hands of their physician.

As shown, the number of clients who decided to get in touch with the lab solely through their physician or directly with the lab at baseline was similar (38.4% through a physician and 35.4% directly with the lab). At T1 and T2, there was a decrease in the number of people who left the management of results and contact with the lab in the hands of their physician (see the first three columns in Fig. 2), and an increase in the number of people having direct contact with the lab for the management of results and health issues, even retaining counseling with their physicians (see second columns in Fig. 2).

**Fig. 3:**
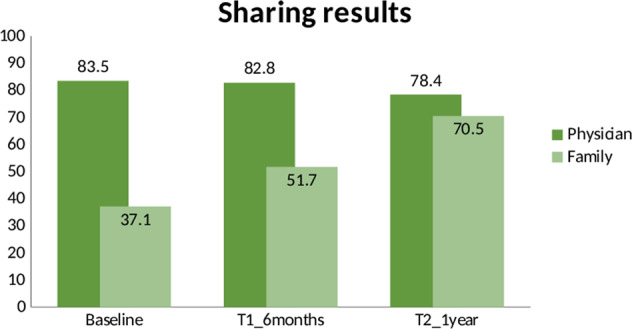
Clients’ preferences in sharing results over time. Shown in the figure are the percentages of participants sharing results with the physician and the family at baseline, at T1 (6 months), and at T2 (1 year).

Finally, at T0, most clients wanted to share their GT results with their physician and only 37% with their family members. At T1, around 82.8% effectively shared results with their physicians and more than 50% with a family member. This last % further increased after 1 year from results, where 70.5% of participants stated that they shared the information within their families (Fig. 3). Furthermore, the choice to share results with their family was not significantly related to the positive or negative outcome of the genetic test, or to the type of genetic test performed (Table S1).

## Discussion

Understanding behavioral changes and decisions related to a genetic test result, and the individual tendencies that induce people to make certain choices, has always been an important question in the context of modern, advanced, and accessible genetic technologies [17, 21, 30–32]. Our contribution provides evidence of the long-term impact of genetic test results and genetic risk communication in a European population, the Italian one, where the market of genetic tests is slowly catching on. In particular, we tried to describe how some health behaviors, risk behaviors, choice of sharing results, and psychological tendencies, including anxiety which is highly debated in the literature, change at 6 months and 1 year after receiving the GT results. As summarized in the “Introduction,” the influence of certain socio-demographic and clinical aspects of our sample of Italian GT users on the intention to change after results have already been discussed in other contributions [26, 27] belonging to the Mind the Risk joint European research program (see “Funding”).

What emerged from our follow-up assessment is that approximately the same percentage of participants who, at baseline, stated that they wanted to change their behavior following GT results, actually did so after 6 months (50% with positive results and 21% with negative results). This percentage increased after 1 year (62.3% positive results and 29.9% negative results), regardless of the positive/negative connotation of results. This would lead us to believe that only people who, from the outset, approach the test with the idea that a genetic predisposition can be “manageable” through their own behavior and who have a high motivation to change, use their results for an effective change of conduct. Support for this idea can also be found in the previous study on the Italian population of GT consumers, which highlighted the presence of high motivation and an internal locus of control (the belief that health is under your own responsibility) in the majority of clients [27].

What is more interesting now is that a high percentage of subjects who had received a negative outcome (meaning that the genetic variant explaining their clinical condition was not found) also preferred to adopt a healthier lifestyle after receiving the results. In particular, our data showed that at 6 months this group of subjects began to have more regular medical checkups, even more frequently than those who had received a positive result (although this trend was not confirmed at 1 year). One hypothesis is that the population approaches genetic tests with the idea of finding an almost unequivocal answer to their clinical condition or family “inheritance,” and if such “confirmation” cannot be found in their genetic makeup, they feel the need to look for an explanation through other screenings. They basically find no answer to their “clinical question,” to their expectations regarding GT results, but this “anxious” search for an unequivocal answer tends to wane over time (after 1 year).

The specific analysis by crossing the type of test performed and the test result, showed that a positive result in GT for food intolerance significantly influenced clients’ changes in lifestyle over time, and a negative result in GT for cancer affected the decision not to change at 1 year from results. These findings suggest that the absence of a known variant that predisposes to the risk of cancer makes people feel erroneously reassured, in light of the fact that a large part of tumors has no genetic origin. This is in line with previous evidence which indicates that “false” reassurance may lead to a reduced adherence to necessary evaluations or surveillance, to ignore other risk factors that contribute to disease, or to engage in potentially negative health behaviors [33–36]. In contrast, a positive genetic test result for food intolerances results in a direct change in health habits (supposedly in their diet).

In a more general analysis relating to the actual long-term behavioral changes adopted by this sample of GT users, a healthier diet and generalized “awareness and attention” toward their own health remain the most mentioned aspects. Physical activity and preventive medical screening remain among the initial intentions and are not confirmed as effective lifestyle changes adopted after 6 months or 1 year.

Overall, risk tolerance and overconfidence do not seem to have a great effect in determining the decision to make lifestyle changes post-GT results, even if at 1 year, people who had a very low risk tolerance resulted in having changed their lifestyle and habits more frequently. This result must be interpreted with caution, due to the fact that we measured participants’ risk attitude with measurements validated in the financial area, and not in healthcare research. As Nicholson et al. [37] argue, risk behavior is patterned: some people have a consistent risk propensity across areas of their life, while others have domain-specific patterns. This implies that risk attitude in one situation is not entirely generalizable to risk propensity in another domain, and personality profiles should be used to predict context-specific risk attitudes and overall risk-taking. Nevertheless, our results can give an indication of whether these variables should be investigated in a genetic risk information field.

Concerning changes over time in psychological tendencies, our data showed that the effect of time after the receipt of GT results reduced anxiety and worry related to one’s health, but at the same time also reduced motivation for health promotion and prevention, health esteem, and positive expectations toward ones’ own health in the future. Such results could mean that GT results do not cause a harmful increase in anxiety, as widely discussed and debated in past literature [38, 39], but induces people to think about their future health condition in slightly more “negative” terms, with the expectation of falling ill in the future. Their positive perception of physical well-being decreases and worryingly also decreases their motivation to avoid the diseases, probably because they believe that they cannot completely avoid health problems in the future because of their genetic makeup. This always leads to a partly deterministic perception of genetic predisposition [20, 40]. Another possible interpretation is that a reduced motivation might simply due to the feeling of having already made the necessary changes (if someone is already eating healthily, their motivation to further change their diet might be pretty low, and therefore they may seek other medical solutions to counterbalance the risk).

Our analyses also revealed that clients who claimed to have actually changed their lifestyle at 6 months had significantly higher levels of anxiety than those clients who made no changes. This is an index which demonstrates that, as already argued in the literature, genetic tests do not cause harmful anxiety since the values do not correspond to clinically worrying values. To the contrary, anxiety in this case can act as a mechanism for lifestyle and health behavior changes [2].

Our data are partially in contrast with what was observed in other studies, for instance that of Bloss et al. [6, 7] who evaluated clients’ reactions 3 and 12 months after GT. This evaluation showed no measurable influence on test-takers in terms of anxiety, use of screening tests among test-takers, nor positive lifestyle changes. Nevertheless, we should consider that the sample from Bloss et al. comprised adults primarily recruited from health and technology companies. These recruits underwent DTC-GT within the first years of its commercialization, so they can be defined as early users of novel technologies and as “lay expert” co-constructors of these techniques [8]. Although early users were moved by their “curiosity” to assess their personal risk susceptibilities or to compare their personal genetic risk factors against their family history of disease, they were also aware of the limited added value of this kind of information for life-altering or lifestyle changing in the absence of a clear clinical application of genetic results. Early adopters of DTC-GT were also motivated by the professional interest in having their personal genome scanned to gain first-hand experience on the product itself. Thus, as observed by McGowan et al. [8] these users fall into a particular category of consumers when compared to our sample of ordinary citizens.

Finally, from the data it emerged that, as regards GT clients’ long-term contacts with the genetic Lab performing the analysis and the decision taken on sharing results, the physician’s professional figure remains important in mediating the management of the result and the possible implications related to health/behavior. In Italy, therefore, a phenomenon of pure DTC-GT does not emerge, since in most cases the doctor is present from the outset as a mediator for the analysis and after some time as a support to interpret results (around 60% of clients approached the test through their physician or with his/her help). As already emerged in the previous study, conducted on all clients enrolled at baseline and currently in press [26], the physician seems to be an important reference figure for managing the genetic results. Despite this, in our current analysis a good percentage of clients preferred to receive their result privately and to be guided by the services offered in a direct relationship with the genetic company (around 35.4% maintained direct contact with GenomaLab). This percentage increased over time if we also consider here the increased number of people who further decided to have “direct contact with the help of physician” at 6 months and 1 year, thus demonstrating a certain desire for freedom in handling the implications of such results [41]. The majority of clients also stated that they shared their results at with the physician (more than 80% if we consider the average of all the different time-points). This demonstrates a frequent shared collaboration between the referred doctor and the patient in genetic decision-making and an attribution of confidence in the doctor.

In this context, however, even if not often mentioned by research participants, the role of the geneticist and genetic counselor should be stressed and further investigated, since their role will be of increasing importance in the future. Indeed, without the appropriate support of geneticists to address the uncertainties that people and families have to manage in the face of a genetic result, the process of coping, adaptation, empowerment, and increase in perceived control toward health could be unsuccessful. Genomics is an area of great development and the role of geneticists and genetic counselors should be optimized.

Finally, with regard to the choice of sharing test results with family members, only a third of participants stated prior to testing that they intended to do so. However, at 6 months more than 50% and at 1 year more than 70% shared results with their families. We wonder whether this choice could be linked to the positive or negative result received, given that the reasons that emerged in past literature for not disclosing results included that test results were uninformative or negative for specific variants [42, 43]. Instead, our results showed that, in the Italian population of GT users, this aspect did not influence participants’ tendency to share their genetic information with family members.

From the literature, we know that there are several other challenges associated with sharing genetic test results within families, including an incomplete understanding of test results, an emotional distance between family members and poor communication skills [44]. Gallo et al. [45] summarized the most common reasons for the disclosure of results within a family, such as a close social relationship with the relative, the need for support, or a perceived need to retrieve information about familial risk. In contrast, the main reasons for non-disclosure within a family included a desire to protect family members from troubling information, a sense of guilt or anxiety, avoidance of negative implications, youth/immaturity or, conversely, the family member’s advanced age. These could have been the reasons which, at baseline, caused participants to be reluctant in their intention to share results within the family, a trend which then reversed after some time. Indeed, as mentioned in “Introduction,” Wöhlke et al. [25] had already reported the tendency of Italian GT users to take responsibility for the family as regards genetic risk information, that is, as a potential weapon for preventing disease onset or other future clinical conditions in the family, and their intention to share results with relatives. Further studies should investigate cultural differences on such aspects of genetic risk communication.

The study’s limitations include the relatively small sample size and lack of gender comparison, due to the predominance of female participants. Another limitation of this research, not specifically connected to the study design, is the lack of a good number of participants who underwent GT for other disease categories (such as Huntington disease or Alzheimer’s). Future research could investigate the relationship between genetic risk awareness and people’s life decisions.

## Conclusions

In conclusion, our primary finding is that people who undergo GT in order to take care of their health become consciously willing to modify their actual behavior, which results in long-term modifications in their behavior following GT results. This indicates that the determination to change is more affected by the subject’s willpower and personal characteristics than by their actual risk level (as determined by the presence or absence of a variant). This evidence should be considered with regard to health-related educational initiatives focused on GT, explaining to individuals that real and persistent changes mainly depend on their willingness to change and not only on the basis of test results. This will also help people to make more informed choices related to genetic issues.

Such studies and results have relevance for the clinical practice, since they allow health professionals (psychologists, geneticists, and counselors) to identify their clients’ personality/psychological aspects, which are to be taken into consideration when proposing genetic services/tests. A deeper knowledge of users’ profiles and decision-making helps health professionals to tailor risk communication and genetic counseling appropriately, in such a way that GT results could be concretely useful for health-related decisions, rather than finishing as unused information [46]. This would also allow the management of the possible impact of results on clients’ lives.

## Supplementary information

Supplemetary material

## Data Availability

The datasets used and/or analyzed during the current study are available from the corresponding author on reasonable request.

## References

[1] Hogarth S, Saukko P (2017). A market in the making: the past, present and future of direct-to-consumer genomics. N Genet Soc.

[2] Oliveri S, Howard HC, Renzi C, Hansson MG, Pravettoni G (2016). Anxiety delivered direct-to-consumer: are we asking the right questions about the impacts of DTC genetic testing?. J Med Genet.

[3] Turrini M (2018). Online genomes: problematizing the disruptiveness of direct-to-consumer genetic tests. Socio Compass.

[4] Charbonneau J, Nicol D, Chalmers D, Kato K, Yamamoto N, Walshe J (2020). Public reactions to direct-to-consumer genetic health tests: a comparison across the US, UK, Japan and Australia. Eur J Hum Genet.

[5] Covolo L, Rubinelli S, Ceretti E, Gelatti U. Internet-based direct-to-consumer genetic testing: a systematic review. J Med Internet Res. 2015;17. 10.2196/jmir.4378. 10.2196/jmir.4378 PMC4704942 26677835

[6] Bloss CS, Schork NJ, Topol EJ (2011). Effect of direct-to-consumer genomewide profiling to assess disease risk. N Engl J Med.

[7] Bloss CS, Wineinger NE, Darst BF, Schork NJ, Topol EJ (2013). Impact of direct-to-consumer genomic testing at long term follow-up. J Med Genet.

[8] McGowan ML, Fishman JR, Lambrix MA (2010). Personal genomics and individual identities: motivations and moral imperatives of early users. N Genet Soc.

[9] Rafiq M, Ianuale C, Ricciardi W, Boccia S (2015). Direct-to-consumer genetic testing: a systematic review of European guidelines, recommendations, and position statements. Genet Test Mol Biomark.

[10] Kalokairinou L, Howard HC, Slokenberga S, Fisher E, Flatscher-Thöni M, Hartlev M (2018). Legislation of direct-to-consumer genetic testing in Europe: a fragmented regulatory landscape. J Community Genet.

[11] Sweeny K, Legg AM (2011). Predictors of interest in direct-to-consumer genetic testing. Psychol Health.

[12] Garg R, Vogelgesang J, Kelly K (2016). Impact of genetic counseling and testing on altruistic motivations to test for BRCA1/2: a longitudinal study. J Genet Couns.

[13] Roberts JS, Gornick MC, Carere DA, Uhlmann WR, Ruffin MT, Green RC (2017). Direct-to-consumer genetic testing: user motivations, decision making, and perceived utility of results. Public Health Genom.

[14] Oliveri S, Ferrari F, Manfrinati A, Pravettoni G. A systematic review of the psychological implications of genetic testing: a comparative analysis among cardiovascular, neurodegenerative and cancer diseases. Front Genet. 2018;9. 10.3389/fgene.2018.00624. 10.3389/fgene.2018.00624 PMC6295518 30619456

[15] Malek J, Slashinski MJ, Robinson JO, Gutierrez AM, Parsons DW, Plon SE (2017). Parental perspectives on whole exome sequencing in pediatric cancer: a typology of perceived utility. JCO Precis Oncol.

[16] Stark Z, Schofield D, Martyn M, Rynehart L, Shrestha R, Alam K (2019). Does genomic sequencing early in the diagnostic trajectory make a difference? A follow-up study of clinical outcomes and cost-effectiveness. Genet Med.

[17] Stewart KFJ, Wesselius A, Schreurs MAC, Schols AMWJ, Zeegers MP (2018). Behavioural changes, sharing behaviour and psychological responses after receiving direct-to-consumer genetic test results: a systematic review and meta-analysis. J Community Genet.

[18] Oliveri S, Renzi C, Masiero M, Pravettoni G (2015). Living at risk: factors that affect the experience of direct-to-consumer genetic testing. Mayo Clin Proc.

[19] Oliveri S, Masiero M, Arnaboldi P, Cutica I, Fioretti C, Pravettoni G (2016). Health orientation, knowledge, and attitudes toward genetic testing and personalized genomic services: preliminary data from an italian sample. Biomed Res Int.

[20] Oliveri S, Pravettoni G (2018). Capturing how individuals perceive genetic risk information: a phenomenological perspective. J Risk Res.

[21] Oliveri S, Pravettoni G. The disclosure of direct-to-consumer genetic testing: sounding out the psychological perspective of consumers. Biol Med. 2016;8. 10.4172/0974-8369.1000316.

[22] Saastamoinen A, Hyttinen V, Kortelainen M, Aaltio J, Auranen M, Ylikallio E (2020). Attitudes towards genetic testing and information: does parenthood shape the views?. J Community Genet.

[23] Bloss CS, Madlensky L, Schork NJ, Topol EJ (2011). Genomic information as a behavioral health intervention: can it work?. Per Med.

[24] Boeldt DL, Schork NJ, Topol EJ, Bloss CS (2015). Influence of individual differences in disease perception on consumer response to direct-to-consumer genomic testing. Clin Genet.

[25] Wöhlke S, Schaper M, Oliveri S, Cutica I, Spinella F, Pravettoni G, et al. German and Italian users of web-accessed genetic data: attitudes on personal utility and personal sharing preferences. Results of a Comparative Survey (n=192). Front Genet. 2020;11. 10.3389/fgene.2020.00102. 10.3389/fgene.2020.00102 PMC7099127 32265977

[26] Oliveri S, Marton G, Vergani L, Cutica I, Gorini A, Spinella F, et al. Genetic testing consumers in Italy: a preliminary investigation of the socio-demographic profile, health-related habits, and decision purposes. Front Public Health. 2020;8. 10.3389/fpubh.2020.00511. 10.3389/fpubh.2020.00511 PMC7578342 33134235

[27] Oliveri S, Durosini I, Cutica I, Cincidda C, Spinella F, Baldi M, et al. Health orientation and individual tendencies of a sample of Italian genetic testing consumers. Mol Genet Genom Med. 2020;8. 10.1002/mgg3.1291. 10.1002/mgg3.1291 PMC7434739 32500972

[28] Prainsack B, Vayena E (2013). Beyond the clinic: ‘direct-to-consumer’ genomic profiling services and pharmacogenomics. Pharmacogenomics.

[29] Masiero M, Oliveri S, Cutica I, Monzani D, Faccio F, Mazzocco K (2020). The psychometric properties of the Italian adaptation of the Health Orientation Scale (HOS). Health Qual Life Outcomes.

[30] Oliveri S, Renzi C, Pravettoni G (2015). Toward an in-depth profiling of DTC users. Clin Genet.

[31] Fanshawe TR, Prevost AT, Roberts JS, Green RC, Armstrong D, Marteau TM (2008). Explaining behavior change after genetic testing: the problem of collinearity between test results and risk estimates. Genet Test.

[32] Stewart KFJ, Wesselius A, Schols AMWJ, Zeegers MP (2018). Stages of behavioural change after direct-to-consumer disease risk profiling: study protocol of two integrated controlled pragmatic trials. Trials.

[33] Yurgelun MB, Hiller E, Garber JE (2015). Population-wide screening for germline BRCA1 and BRCA2 mutations: too much of a good thing?. J Clin Oncol.

[34] Linderman M, Nielsen D, Green R (2016). Personal genome sequencing in ostensibly healthy individuals and the PeopleSeq Consortium. J Pers Med.

[35] Becker F, Van El CG, Ibarreta D, Zika E, Hogarth S, Borry P, et al. Genetic testing and common disorders in a public health framework: how to assess relevance and possibilities. Eur J Hum Genet. 2011;19. 10.1038/ejhg.2010.249. 10.1038/ejhg.2010.249 PMC3327518 21412252

[36] Oliveri S, Scotto L, Ongaro G, Triberti S, Guiddi P, Pravettoni G (2019). “You do not get cancer by chance”: communicating the role of environmental causes in cancer diseases and the risk of a “guilt rhetoric”. Psychooncology.

[37] Nicholson N, Soane E, Fenton-O’Creevy M, Willman P (2005). Personality and domain-specific risk taking. J Risk Res.

[38] Hamilton JG, Lobel M, Moyer A (2009). Emotional distress following genetic testing for hereditary breast and ovarian cancer: a meta-analytic review. Heal Psychol.

[39] Heshka JT, Palleschi C, Howley H, Wilson B, Wells PS (2008). A systematic review of perceived risks, psychological and behavioral impacts of genetic testing. Genet Med.

[40] Dar-Nimrod I, Heine SJ (2011). Genetic essentialism: on the deceptive determinism of DNA. Psychol Bull.

[41] Oliveri S, Pravettoni G, Fioretti C, Hansson MG (2016). Let the individuals directly concerned decide: a solution to tragic choices in genetic risk information. Public Health Genom.

[42] Tercyak KP, Peshkin BN, DeMarco TA, Brogan BM, Lerman C (2002). Parent–child factors and their effect on communicating BRCA1/2 test results to children. Patient Educ Couns.

[43] Hughes C, Lerman C, Main D, Peshkin BN, Wenzel L, Narod S (2002). All in the family: evaluation of the process and content of sisters’ communication about BRCA1 and BRCA2 genetic test results. Am J Med Genet.

[44] Daly MB, Montgomery S, Bingler R, Ruth K (2016). Communicating genetic test results within the family: is it lost in translation? A survey of relatives in the randomized six-step study. Fam Cancer.

[45] Gallo AM, Angst DB, Knafl KA (2009). Disclosure of genetic information within families: how nurses can facilitate family communication. Am J Nurs.

[46] Gorini A, Pravettoni G (2016). Why do we pay for information that we won’t use? A cognitive-based explanation for genetic information seeking. Eur J Hum Genet.

