# Supplementary material for: What people really change after genetic testing (GT) performed in private labs: results from an Italian study
Source: Eur J Hum Genet. 2021 Apr 12;30(1):62–72. doi: 10.1038/s41431-021-00879-w (PMC8738765; doi:10.1038/s41431-021-00879-w)
Supplement: Supplementary file 1 — Supplemetary material [file 41431_2021_879_MOESM1_ESM.docx]

**Supplementary Material**

*Lab description*

GenomaLab is a private molecular genetics laboratory, providing diagnostic support to public and private health clinics, hospitals, medically-assisted procreation centres and physicians specialized in different fields. The lab also provides a wide choice of personalized genetic services for private customers, who can decide to approach the laboratory through their physician or by contacting the Lab and its geneticists (analysis by categories https://www.laboratoriogenoma.eu/eng/analyses_list.asp). Based on the requests, Genoma provides specific packages for analysis of variants or a personalized set of gene testing, tailored on users’ clinical condition, medical verification and family history of disease. The price range for testing in this Lab varies from a minimum of EUR 80–100 for genetic lifestyle analysis, specific intolerance, or analysis of specific variants related to a single disease, to EUR 450–500 for a predictive package analysis for a series of diseases (e.g., different types of cancer). The Lab frequently collaborates with clinicians from different medical contexts, such as gynecologists, nutritionists, oncologists and family physicians. This does not exclude the possibility of accessing services by directly referring to lab professionals.

All the analysis are carried out according to the Good Laboratory Practice (GLP) requirements imposed by European standards. In addition to the ISO 9001 scheme certification, the lab possesses an ISO/IEC 17025: 2017 accreditation issued by the Italian regulatory body ACCREDIA, with an equivalent function as the CMS (United States Department of Health & Human Sevices Center for Medicare & Medic Aid Services) which issues CLIA (Clinical Laboratory Improvement Amendments) certifications in the US. Genomalab is currently seeking to obtain the ISO 15189 accreditation, which is the European equivalent to the American CLIA.

Genomalab is also included in the list of AIFA (Agenzia Italiana del Farmaco) phase 1 laboratories and is accredited with the regionals health care systems (equivalent to GLP certification). In the end, the analysis that are performed by Genoma laboratory follow CE-IVD protocols with external annual quality assurance checks carried out with accredited international circuits 17043.

*Procedure*

Clients who underwent genetic testing for food intolerances, disease susceptibility, disease carriers and reproductive purposes were included in the study. Researchers explained to participants the purposes of the study, the study procedure, the follow up monitoring, and were invited to sign in a written informed consent form. They filled in *ad hoc* questionnaires at three-points in time: T0-before genetic testing, T1-after six months from results and T2-after one year from results. Data were collected using the Survey Monkey platform, an open source online survey application which enables users to develop and publish online surveys and register responses (www.surveymonkey.com).

Ethical approval was obtained from the Institutional Review Board at the University of Milan (the principal coordination center of the survey), and by the Centre for Research Ethics and Bioethics, University of Uppsala (coordinator of the Mind the Risk Project; see funding declaration).

*Measures*

Clients completed a structured and self-administered ad hoc questionnaire that could be divided into different sections.

- *Socio-demographic variables:* the questionnaire contained a set of specific questions aimed at investigating the sociodemographic characteristics of the respondents, such as gender, age, educational level, marital status, parenthood, and current employment.
- *Clinical experiences:* the questionnaire contained a set of questions designed to gather information on participants’ clinical experiences, such as past and/or current diseases (Have you suffered/are you currently suffering from specific diseases?), familial disease and genetic history (In your family history, is there a relevant illness experience? Do you have a family history of hereditary/genetic disease?) with “Yes”, “No”, “I do not know” as answer options.
- *Lifestyle and health-related risk behaviors:* the questions in this section investigated daily respondent habits, such as health screening behavior (How regularly do you undergo a medical checkup? Multiple choice answers “yes, regularly”, “when I have symptoms”, “sometimes”, “very rarely”, “no, never”), type of diet (“Which of these food categories do you predominantly consume? Choose 3 options” followed by the list of all the existing food categories, except insects), and physical activity (investigated through the International Physical Activity Questionnaire - Short Form)^1^. Specific questions were included to investigate smoking behaviors (Are you a smoker or former smoker?) and dietary risk behaviors. For this last measure, participants had to indicate how often they had incorrect dietary habits on a 5-point Likert scale, from 0 “Never” to 4 “Daily”, based on the following items: 1) eat fast food; 2) eat junk food during happy hour; 3) eat sandwiches; 4) eat fried food; 5) eat dessert/cake/chocolate; 6) drink soda; 7) drink alcohol. An overall score of incorrect dietary habits were calculated with the sum of the scores assigned to each item.
- *Motivation for testing and perceived utility:* participants were asked to exactly report which kind of testing they underwent, their motivation for undergoing the genetic panel (open ended questions), and their idea of the implications for future health-related behaviors and decisions (e.g "Do you think your lifestyle will change after receiving the result of the genetic analysis?", with “yes” or “no” answer options, followed by “If yes, how?” and a list of items for possible changes as represented in Figure 1), and finally, the willingness to share results with other people (family, physicians and others).
- *Health Orientation Scale* (HOS)^2^ subscales: The HOS is a self-reporting questionnaire developed by Snell and colleagues (1991) with the aim of assessing personality traits associated with health behaviors. The original version was composed of 50-items, divided into ten subscales. The scale was validated in the Italian context by Masiero *et al.* (2020)^3^; the Italian adaptation consisted of 36 items, grouped into seven dimensions. In this follow up protocol, four subscales in the HOS Italian adaptation were submitted to participants: 1) *Motivation for health promotion and prevention* (MHPP; 9-items)(score ranging from 0 to 36) which represents people’s tendency to actively avoid risky behaviors in order to protect their well-being and physical health; 2) *Health Esteem (HES*;7 items)*,* which describes people who feel positive and confident in handling their current health status and future physical condition (score ranging from 0 to 32); 6) *Health Anxiety (HA)* (4 items) which identifies people whose negative feelings influence their health perception (score ranging from 0 to 16)*; 7) Health Expectations (HE)* (2 items)*,* which describes people with positive expectations for their future health (score ranging from 0 to 8). Participants must indicate the extent to which each statement (item) of the subscales reflects their profile on a 5-point Likert scale (0 = not at all characteristic of me, 1 = slightly characteristic of me, 2 = somewhat characteristic of me, 3 = moderately characteristic of me, and 4 = very characteristic of me). Total scoring for each subscale is the sum of the points assigned to the corresponding items.
- *Overconfidence*: We asked participants to assess their overall risk-taking attitude in a financial setting and, in the analysis, we tried to draw a parallel with their health attitude. We submitted a one item scale retrieved from the contribution of Pan and Stateman: *Some people believe that they can pick stocks that would earn higher-than-average returns. Other people believe that they are unable to do so. Please indicate your belief by circling the number on a scale ranging from “Strongly believe I cannot pick higher-than-average stocks” to “Strongly believe I can pick higher-than-average stocks*”^4^. High scores indicate participants’ higher confidence in taking very high risks.
- *Health and Retirement Study (HRS)****:*** The Health and Retirement Study^5^ is a scale that explores risk tolerance, that is, the amount of risk and uncertainty that a person is able to handle. Risk tolerance is assessed through the following scenario: “*Suppose that you are the only income earner in the family, and you have a good job guaranteed to give you your current (family) income every year for life. You are given the opportunity to take a new and equally good job, with a 50–50 chance it will double your (family) income and a 50–50 chance that it will cut your (family) income by a third. Would you take the new job?* Individuals accepting this new, risky job are then required to consider one with a higher downside risk: *Suppose the chances were 50–50 that it would double your (family) income, and 50–50 that it would cut it in half. Would you still take the new job?* Those initially declining the new job consider one with a lower downside risk: *Suppose the chances were 50–50 that it would double your (family) income and 50–50 that it would cut it by 20 percent. Would you then take the new job?* These two responses categorise individuals into four categories: *unwilling to risk a one-fifth income cut* [Very low risk tolerance], *willing to risk at most a one-third cut* [Low risk tolerance], *willing to risk a one-third to a one-half cut* [Medium risk tolerance], and *willing to risk at least a one-half cut* [High risk tolerance]”.
- *Lab contact and decision on sharing results:* in this section, participants were asked to indicate how they kept in touch with GenomaLab (multiple choice question with the following options: 1) through the physician as an intermediary (which means having entrusted the doctor to retain contact with the lab for the analysis and receipt of results); 2) autonomously but with a physician’s counselling (which means having maintained direct contact with the Lab for analysis and receipt of results, but was monitored by the doctor for the procedure and interpretation of result; 3) directly with the genetic lab (the entire process, from analysis to receipt of results, was managed independently using the counselling services offered by the laboratory; 4) other.

*Data Analysis*

Descriptive statistics (frequencies and/or mean and standard deviation scores) were calculated to report socio-demographic data and clinical aspects at baseline, such as clients’ clinical history, type of genetic testing required, GT results and the preferred Lab contact and decision on sharing results. Frequencies and percentages were also calculated for variables related to participants’ lifestyles such as diet or physical activity. Mean and standard deviations were calculated to explore behaviors and psychological tendencies of GT users at baseline (risky eating behaviors, physical activity, medical checkups, intention to change lifestyle, health orientation, overconfidence, and risk tolerance).

One-way repeated measures analysis of variance (ANOVAs) were conducted to evaluate changes over time in risky eating behaviors and psychological tendencies (HOS subscales). Furthermore, we performed a mixed factorial analysis of variance (ANOVA) to assess the changes at T1 and T2 in HOS subscales, based on the reason for the clients’ genetic testing (infertility problems, intolerance, cancer susceptibility) or GT results (positive vs negative). The Bonferroni post hoc test was used to determine which groups were significantly different.

Contingency tables and Chi-square tests were performed to compare the groups of users who received a positive vs negative GT result with their risk tolerance, medical check-up habits and the decision to share GT results with the family. Expected values ​​and residuals in every box were calculated, in order to verify if a specific group gave a significantly higher or lower rate of response (observed values) to certain items, compared to the expected percentage calculated on the number of recruited subjects. In the interpretation of the standardized residuals, 1.96 was considered to be the discriminant value for a confidence level of 95%.

We used *SPSS* (Version 26) statistical software analysis package for statistical analysis.

*Results*

*Table S1. Non-significant comparisons.*

| **Comparison and results** | **Type of test** | **Values** | **Significance** |
| --- | --- | --- | --- |
| People who received a positive result reported that they had modified their lifestyle after 6 months, and this percentage increased after 1 year (non-significant trend). | McNemar’s test | T1: 50.8%; N=31  T2: 62.3%; N=38 | p= .065 |
| Among people who received negative results, 21% changed their lifestyle after 6 months, and the percentage increased after 1 year (non-significant trend). | McNemar’s test | T1: 21%; N=8  T2: 28.9%; N=11 | p= .625 |
| “Healthier diet” was the most effectively adopted behavior both at 6 months and at the 1 year follow up (non-significant trend) | Cochran Q test | T1: 76.5%; N=75  T2: 79.5%; N=78  X^2^(2) =.167 | p = .920 |
| “Overall health consciousness” was among the adopted behaviors both at 6 months and at the 1 year follow up (non-significant trend). | Cochran Q test | T1: 58.8%; N=58  T2: 51%; N=50  X^2^(2) =1.714 | p =.424 |
| Difference in the frequency of medical checkups based on the type of GT performed. No significant changes were found after 6 months and 1-year. | Chi quare | T1: X^2^(8) = 12.718;  T2: X^2^(8) = 5.915; | p =.122  p =.657 |
| Choice to share results with the family not influenced by positive/negative results of the genetic test. | Chi square | T1: X^2^(1) = .689;  T2: X^2^(1)= .42; | p =.407  p =.517 |
| Choice to share results with the family not influenced by the type of genetic test performed. | Chi square | T1: X^2^(1) = .784;  T2: X^2^(1) = .461; | p =.321  p =.537 |

**References**

1 Lee PH, Macfarlane DJ, Lam T, Stewart SM. Validity of the international physical activity questionnaire short form (IPAQ-SF): A systematic review. *Int J Behav Nutr Phys Act* 2011; **8**: 115.

2 Snell WEJ, Johnson G, Lloyd PJ, Hoover MW. The health orientation scale: A measure of psychological tendencies associated with health. *Eur J Pers* 1991; **5**: 169–183.

3 Masiero M, Oliveri S, Cutica I, Monzani D, Faccio F, Mazzocco K, *et al.* The psychometric properties of the Italian adaptation of the Health Orientation Scale (HOS). *Health Qual Life Outcomes* 2020; **18**: 69.

4 Pan CH, Statman M. Questionnaires of Risk Tolerance, Regret, Overconfidence, and Other Investor Propensities. *SSRN Electron J* 2012. doi:10.2139/ssrn.1549912.

5 Barsky RB, Juster FT, Kimball MS, Shapiro MD. Preference Parameters and Behavioral Heterogeneity: An Experimental Approach in the Health and Retirement Study. *Q J Econ* 1997; **112**: 537–579.
